# An Adapted Behavioral Framework for Integrating LGBT+ in Dental Curriculum: Learner‐Centered Training to Person‐Centered Care

**DOI:** 10.1002/jdd.13887

**Published:** 2025-03-28

**Authors:** Abbas Jessani, Alexia Athanasakos, Tamanna Tiwari

**Affiliations:** ^1^ Department of Dentistry Schulich School of Medicine and Dentistry Western University London Ontario Canada; ^2^ Department of Epidemiology and Biostatistics Schulich School of Medicine and Dentistry Western University London Ontario Canada; ^3^ Department of Community Dentistry and Population Health School of Dental Medicine University of Colorado Anschutz Medical Campus Aurora Colorado USA; ^4^ Centre for Oral Disease Prevention and Population Health Research School of Dental Medicine University of Colorado Boulder Colorado USA

**Keywords:** dental education, LGBT+ people, oral health needs, person‐centered care, sexual and gender minorities, social determinants of health

## Abstract

Lesbian, gay, bisexual, transgender, or other sexual orientations and gender identities (LGBT+) people report poorer oral health outcomes compared to their heterosexual and gender‐binary counterparts due to social and structural inequities. As such, there is a need for robust integration of social determinants of health (SDOH) and their intersectionality with oral health among LGBT+ people. An SDOH framework was adapted, based on education, organization, and community domains, to integrate the LGBT+ teaching and content into already established dental curricula. The *education* domain emphasizes the integration of didactic and experiential education to address the person‐centered oral health needs of sexual and gender minorities. This includes didactic content delivery by LGBT+ people and representation from diverse gender and sexual backgrounds in case‐based learning and community service‐learning. The *organization* domain encourages the embedment of health equity and the development of inclusive environments supportive of gender and sexual minorities into the mission statements of dental schools and the continuing professional development. Important measures include the integration of preferred pronouns at all levels of the organization, diverse gender representation on patient intake forms, and dedicated safe spaces for all minorities, including sexual and gender minorities. Lastly, the *community* domain emphasizes the development of partnerships between LGBT+ community organizations and dental schools to develop community‐integrated educational models for the teaching of SDOH and the addressal of unmet LGBT+ oral health needs. Integrating this adapted SDOH framework will provide learners, faculty, and staff with a comprehensive understanding of the person‐centered needs of LGBT+ community members. This will encourage learners to approach gender and sexual minorities with empathy and cultural humility while providing trauma‐informed, person‐centered care.

## 1 Introduction

Oral health disparities that adversely affect lesbian, gay, bisexual, transgender, or other sexual orientations and gender identities (LGBT+) people persist [1, 2]. (The authors recognize that terminology and acronyms within this population are continuously evolving; however, for this paper, the acronym LGBT+ will be used.) Compared to their heterosexual and gender‐binary counterparts, LGBT+ people are more likely to avoid and mistrust healthcare providers due to previous or anticipated stigmatization and/or discrimination during healthcare encounters [1]. These disparities are compounded by social and structural inequities that adversely impact the oral health outcomes of LGBT+ people [1, 3].

LGBT+ curricular content in North American dental schools has been reported to be limited. A study conducted by Hillenburg et al. [4] revealed that 29% of dental schools in the United States reported no LGBT+‐covered content—and of the 71% with coverage—only 3.68 h were dedicated to LGBT teaching. Similarly, a recent study by Jessani et al. [5] revealed gaps in sexual and gender minority‐related dental curricula, including a robust delivery of social determinants of health (SDOH) in Canadian dental schools [5]. Oral health disparities pertinent to alcohol, tobacco, or other substance use; pediatric and adolescent oral health issues; and sex reassignment surgery were sparsely taught in the dental curriculum, potentially translating into inadequate trauma‐informed, person‐centered care concerning LGBT+ people [4, 5]. Therefore, integrating the LGBT+ curriculum within the framework of SDOH is warranted [5]. This requires explicit guidelines that consider SDOH and the interconnected nature of oral health to ensure equity and person‐centered care for LGBT+ people. The SDOH framework by Tiwari and Palatta [6] supports the incorporation of SDOH into predoctoral dental curricula using threedomains: education, organization, and community. The *education* domain emphasizes the prevention of isolated learning environments by encouraging the integration of didactic and clinical education to facilitate the exposure of learners to diverse populations [6]. The *organization* domain highlights the importance of embedding the values of health equity into the mission statements of organizations and the continuing professional development regarding the advancement of SDOH knowledge [6]. Lastly, the *community* domain emphasizes the development of partnerships between communities and dental schools for the shaping of learners’ understanding of SDOH [7].

**FIGURE 1:**
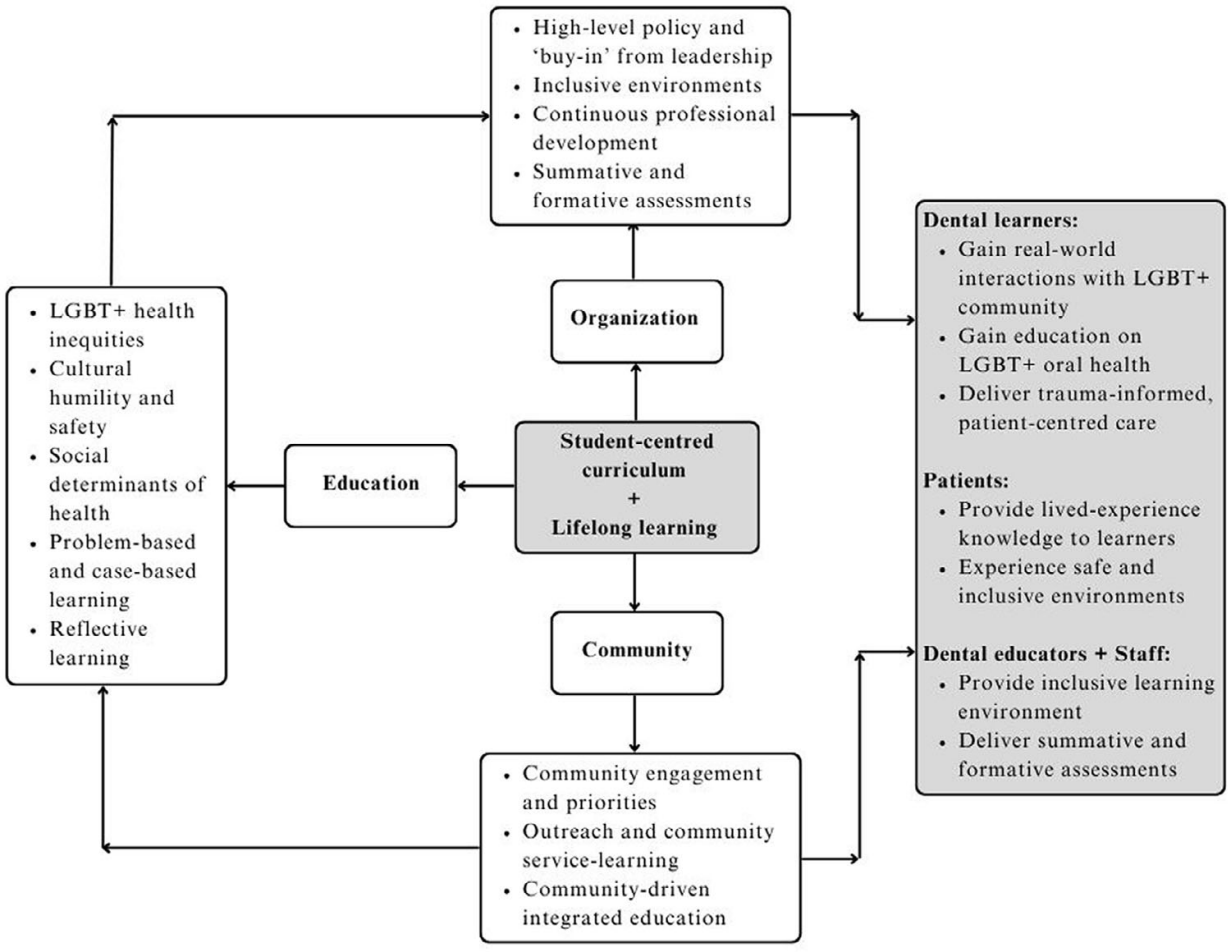
Adaptation of the SDOH framework integrates the needs of LGBT+ people into the dental curriculum.

Given the lack of literature reporting a streamlined approach for the training of learners on the oral health needs of LGBT+ people and the minimal emphasis on the challenges of incorporating sexual and gender minorities into dental curricula, the aim of this perspective paper is to present an adapted SDOH framework that integrates the LGBT+ in all the facets of dental curricula (Figure 1).

## 2 Framework

This adapted framework will showcase how progressive efforts can be utilized in dental schools to facilitate the incorporation of LGBT+ needs into their existing curricula. These domains are not to be used in isolation but rather as a whole, with one assisting the other.

### 2.1 Domain 1: Education

Despite recognizing the need to incorporate LGBT+‐specific training and content in dental curricula, several challenges have been reported in papers by Hillenburg et al. [4] and Jessani et al. [5]. These challenges include saturated curricula within both foundational and clinical oral health sciences, which leaves limited room for the implementation of behavioral content (including LGBT+‐specific topics); limited time available for the delivery of dental content; insufficient knowledge about the implementation of LGBT+‐specific teaching; a lack of relevant faculty experience; insufficient available funding; and a lack of perceived importance. However, sensitivity to and respect for the cultural identity of LGBT+ people is essential for optimal oral health care delivery and, therefore, requires robust changes in dental curricula.

#### 2.1.1 Integration of LGBT+ Within the SODH Curriculum

The LGBT+ community faces unique social and behavioural challenges due to pervasive stigma and discrimination, social pressures, sexual behaviors, prejudice, *cis*‐heteronormativity, and negative encounters with healthcare providers who lack the following: gender‐ and sex‐inclusive practices, Indigenous cultural safety, and experience in directly treating sexual and gender minorities [5]. The substantial representation of LGBT+ people from visible minorities further exacerbates their experiences as gender and sexual minorities, creating a “minority within a minority” [1]. This dual marginalization impacts their cultural safety and social acceptance [1].

Although there is no ideal case study on LGBT+ training in the dental curriculum, Haley et al. [8] have emphasized a baseline level of knowledge and sensitivity to meet the standards and competencies of both the Commission on Dental Accreditation (CODA) and the Association of Canadian Faculties of Dentistry (ACFD) and improve the overall understanding of the LGBT+ population within the context of oral health. Dental schools should, at minimum, reduce LGBT+‐related bias by delivering content with the intent of their learners’ understanding the following: (1) sexual orientation, gender identity, and LGBT+ terminologies; (2) self‐perceived and clinical health needs and risks; (3) impacts of LGBT+‐related discrimination and health inequities; and (4) provision of inclusive care [7].

LGBT+‐specific topics can effectively increase knowledge of  healthcare problems and improve the comfort levels of learners working with LGBT+ patients [7]. Morris et al. [7] proposed a three‐part didactics, discussion, and delivery strategy. During didactic teaching, information related to terminology, health inequities, and the essentials of inclusive care may occur [7]. Small group discussions should be implemented to encourage the application of knowledge to LGBT+‐specific case scenarios and address individual biases [7]. Finally, learners should be provided the opportunity to have “real‐world” interactions by delivering care to members of the LGBT+ community in the form of outreach and service‐learning [7, 9]. In a 2023 study by Salter et al. [10], the introduction of an LGBT+ competency course to dental learners ranging from D1 to postdoctoral residency proved to be an effective method of improving learners’ awareness and confidence in treating LGBT+ patients. For instance, after the training, results showed a 13% increase in the number of learners who felt clinically prepared to treat LGBT+ patients, a 32% increase in those who reported improved knowledge of LGBT+ health disparities, and a 10% decrease in bias towards gender and sexual communities [10].

Attention should be given to the professional development and training of the faculty, as they are responsible for providing fulsome training to the learners, including the issues pertinent to sexual and gender minorities. Healthcare providers with expertise in LGBT+, including physicians and nurses, along with LGBT+‐identified community members with lived experience, must be engaged in “training the trainer” [11].

To assist faculty with their teaching of LGBT+ inequities in health, the following learning objectives should be considered: (1) examining the provider's attitudes for implicit biases (i.e., stereotyping, assuming heterosexuality, or cisgender identity) and assessing the patient's trust in their provider; (2) assessing the extent of LGBT+ health disparities; (3) teaching cross‐cultural communication skills (i.e., using gender‐neutral and inclusive language); (4) ensuring communication is at a health literacy level appropriate for the patient; (5) effectively using and selecting culturally competent translators; and (6) fostering heightened empathy for patients (i.e., recognizing LGBT+ health disparities, recognizing diverse identities, and validating the feelings of patients) [4]. To the authors’ knowledge, a lack of literature pertaining to the implementation of such learning objectives is present within the curriculum of dental, medical, and nursing schools, with many studies primarily discussing temporal aspects of the LGBT+ curriculum while presenting a solid implementation and integration that translates to person‐centered care [10, 12].

#### 2.1.2 Uniqueness of Diverse Populations vs. Oral Health Needs

In clinical settings, gender and sexual identity are almost always centered around the historical, non‐inclusive LGBT acronym, which furthers the risk of excluding Two‐Spirit, questioning or queer, intersex, nonbinary, or asexual members or misclassifying others who identify as members of the “+” category in LGBT+ [1, 3]. While current curricula pertaining to gender and sexuality have the potential to heighten one's awareness of LGBT needs, focusing on addressing the unique needs of the (sub)populations within the LGBT+ acronym is rare [3]. As such, it is important to consider *how* LGBT+ identities and experiences are portrayed within the curriculum by integrating varied representations of LGBT+ communities rather than a mere universal “LGBT+ experience” [13]. As a foundational step, the definitions of LGBT+ concepts and terms include Two‐Spirit, nonbinary, intersex, queer, questioning, sexual orientation, gender identity, and so forth to showcase that gender and sexuality are spectrums that evolve over time [13]. A culture of open dialogue and expression is promoted, enabling patients and learners to freely express their gender identities, including the use of their preferred pronouns [1].

Dental curricula materials should promote a culture of inclusion by recognizing the LGBT+ population as a group with diverse healthcare and oral health needs. Within the SDOH framework, contextual training on LGBT+ oral health should be provided. The intersectionality of poverty, race and ethnicity, and social identity—along with gender and sexual identity—should be meaningfully integrated into the curriculum.

#### 2.1.3 Diversification of Teaching Methodologies

Although lecture‐based teaching, which is typically used to deliver LGBT+‐specific content, can lead to the accomplishment of learners’ educational objectives and goals, it cannot adequately enable the development of “superior professional skills” among learners [5, 14]. As such, the incorporation of experiential and small‐group learning (including problem‐based learning and case‐based learning), simulated patients, and community service‐learning rotations into the dental curriculum can more realistically engage diverse patient populations [14].

#### 2.1.4 Cultural Humility and Safety

It is also imperative that LGBT+ people from a broad cross‐cultural range, including members of various racial, ethnic, and religious backgrounds, as well as those with diverse refugee and socioeconomic statuses, experience an environment characterized by safety and inclusivity within the oral health delivery system [15]. To ensure that members of the LGBT+ community feel safe, dental schools—especially their dental clinics—must foster an inclusive environment by incorporating LGBT+ symbols and materials and using inclusive, gender‐neutral language and pronouns [15]. The language used in patient education materials, as well as spoken to the patient directly, should be gender‐neutral and inclusive to all patients [15]. Learners should be taught the importance of avoiding assumptions and inferring judgment when selecting inclusive language. For example, rather than mother/father, terms like parent/guardian can be used. By providing learners with an insight into a “safe environment” for LGBT+ people during these formative learning years, learners can apply these practices in “real‐world” dentistry upon graduation.

However, the implementation of inclusive environments in dental schools faces several challenges, the most common being a lack of faculty “buy‐in” and preparedness as well as inadequate resources and institutional policies [5, 16]. Faculty members may find themselves struggling to navigate the complexities of adapting their teaching methods to integrate the diverse needs of LGBT+ people, particularly when gaps in knowledge among faculty exist and policies fail to promote strategies geared towards fostering an inclusive environment for not only LGBT+ patients but also learners, faculty, and staff [5, 16].

#### 2.1.5 Integrated Reflection Exercises and Evaluation

In dental curricula, reflective learning mostly consists of writing; however, to accommodate diverse learning preferences, dental schools should integrate various forms of reflection, including verbal and audio recordings and storytelling and incident‐based narratives, depending on the course objectives [17, 18]. Creative reflection approaches may foster learners’ sense of belonging and support interpersonal skills and personal development [17]. Competencies related to LGBT+ and minority populations should be integrated into assessments, emphasizing professionalism, ethics, and trauma‐informed, person‐centered care to uphold the highest standards of best practices.

### 2.2 Domain 2: Organization

These subdomains represent the role of dental schools and community organizations that provide support to curricular efforts.

#### 2.2.1 High‐Level Policy and “Buy‐In” From Leadership

Creating a safe and inclusive environment for LGBT+ people requires a well‐defined, high‐level institutional policy and a strong “buy‐in” from the dental school's executive leadership committees. This should include at least one champion dedicated to equity, diversity, and inclusion and LGBT+ inclusion efforts within the dental school (i.e., explaining the goals and rationale for developing safe environments, scheduling professional development sessions, etc.) as the commitment and agreement of one member helps raise awareness and create “buy‐in” amongst the others. Policies should advocate for health equity for LGBT+ populations within a curriculum that is designed to be “spirally” integrated. All policies, no matter the degree of complexity, must be endorsed by the leadership committee to be integrated—this includes inclusive policies and practices such as the integration of pronouns on patient intake forms.

#### 2.2.2 Inclusive Environments

Upon entering healthcare environments, such as preclinical labs and dental clinics, steps should be taken to ensure a safe, affirming, and inclusive environment for LGBT+ people. Checklists can be effective for ensuring that policies, statements, forms of communication, and patient intake forms are inclusive of gender identities across the gender diversity spectrum. The following are example questions that can be included: (1) Is gendered information included only when critical to the case or patient care; otherwise, are terms such as person, patient, or individual used? (2) Is gendered terminology used accurately and specifically? (3) Are case scenarios and/or patient presentations conveyed without the promotion of stereotypes? [19]. Information on pronouns and names should also be collected to allow patients and learners the opportunity to indicate how they wish to be addressed. Such information can be collected verbally and on the patient intake form and can be printed on the name tags of learners. Within the waiting room, oral health education materials should integrate LGBT+ symbols, diverse gender expression, same‐gender and ‐sex couples, and LGBT+ families. Brochures and resource materials from local LGBT+ organizations should be offered as a means of support.

#### 2.2.3 Evidence‐Informed Care

Available literature highlights an overall dissatisfaction with the level of LGBT+ content covered in North American dental schools, with many relevant topics such as alcohol, tobacco, or other substance use, pediatric and adolescent oral health, and sex reassignment surgery issues either not included or unknown to the majority of participating dental schools [4, 5]. To effectively integrate LGBT+ content into the dental curriculum, two key components must be considered: (1) integrating existing evidence‐based practices pertinent to the needs of LGBT+ people and (2) implementing community engagement with relevant organizations to conduct needs assessments and community‐based interventions pertinent to the LGBT+ community. The former component involves implementing strategies such as cultural sensitivity training for learners, faculty, and staff (i.e., understanding potential dental care needs specific to LGBT+ people, such as hormonal effects on oral health in transgender patients), using gender‐affirming language and practices (i.e., using gender‐neutral, inclusive, and open‐ended language), and adopting trauma‐informed care approaches (i.e., delivering nonjudgmental care through understanding and empathy for LGBT+ patients) [1, 5]. These strategies should be incorporated into the didactic and experiential curriculum to ensure the delivery of evidence‐informed care.

### 2.3 Domain 3: Community

Meaningful partnerships between academic institutions and the community are essential not only for enhancing learners’ education but also for addressing the unmet oral health needs of LGBT+ community members. Through the dynamic process of guidance, support, and codevelopment, interactions between learners and community stakeholders can encourage the gradual awareness and understanding of behaviors, norms, and practices characteristic of the community.

#### 2.3.1 Community Engagement and Priorities

Community engagements should be considered an integral component of didactic delivery. Facilitated discussions with LGBT+ community stakeholders and community members with lived experience can ensure that the developed curriculum is not only sensitive to the needs, perspectives, and priorities of LGBT+ people but also avoids the perpetuation of biases. In North America, a significant portion of the LGBT+ population consists of immigrants from diverse ethnic and socioeconomic backgrounds, including countries with anti‐LGBT+ laws. Jessani et al. [5] have suggested that a lack of community‐based research serves as a barrier to the incorporation of appropriate LGBT+ content into dental curricula. With more queer‐identifying people immigrating to North America, learners must understand the risk‐based oral health needs of the LGBT+ population while being broadly aware of SDOH impacting oral disease prevalence.

Leadership within the dental schools and community stakeholders should have a collaborative partnership, cocreating the curricular objectives to adequately reflect the needs of LGBT+ and other minorities. Implementing strategies that increase power‐sharing and trust‐building can enhance the relationship between LGBT+ patients and providers, while also helping to prevent the perpetuation of stereotypes, prejudice, or discriminatory attitudes.

#### 2.3.2 Outreach and Community Service‐Learning

Through experiential and service‐learning placements, learners can engage with the LGBT+ populations and learn about their lived experiences with stigma, discrimination, bullying, violence, traumas, and other related issues. This hands‐on approach encourages learners to address barriers to care and develop inclusive, trauma‐informed, person‐centered practices that promote equity and accessibility for LGBT+ populations [9].

## 3 Recommendations and Future Directions

The following recommendations are intended to guide the enhancement of LGBT+‐specific content in dental schools and the cultural awareness of learners, faculty, and staff:

1. Didactic teaching on SDOH, emphasizing cultural humility and trauma‐informed, person‐centered care, and sexual and gender diversity, into didactic and experiential phases of the dental curriculum.
2. Establishment of meaningful partnerships with local LGBT+ community organizations to provide learners with community service‐learning opportunities.
3. Collaboration with accreditation bodies (e.g., CODA, ACFD) to develop integrated competencies for the delivery of person‐centered dental care for LGBT+ people.
4. Training of personnel in direct contact with LGBT+ people, such as administrative staff, dental assistants, and dental hygienists, on cultural sensitivity, including appropriate use of pronouns and preferred names.
5. Implementation of ongoing professional development for educators and practitioners to support continuous knowledge growth and cultivate an inclusive learning environment for learners and diverse populations.
6. Diversification of learners and educator population to include meaningful representation of sexual and gender minorities.
7. Enhancement of logistical support to oversee the implementation and ensure sufficient financial resources are allocated for the maintenance of high‐quality LGBT+ curricula.
8. Prioritization of community‐based research for the development of risk‐based oral health interventions tailored to the needs of LGBT+ populations.

## Author Contributions

All authors contributed equally to the manuscript.

## Ethics Statement

The authors have nothing to report.

## Conflicts of Interest

The authors declare no conflicts of interest.

## Data Availability

The authors have nothing to report.
